# Improving the Assessment and Management of Constipation in Elderly Inpatients: A Quality Improvement Project in a Geriatric Ward

**DOI:** 10.7759/cureus.116396

**Published:** 2026-09-16

**Authors:** Benyam G Yirdaw, Humna Anis Shaikh, Victor Adekiya

**Affiliations:** 1 General Medicine/Acute Medicine, Fairfield General Hospital, Bury, GBR; 2 Internal Medicine, Fairfield General Hospital, Bury, GBR; 3 Geriatrics, Fairfield General Hospital, Bury, GBR

**Keywords:** 6-point checklist, constipation, frailty syndrome, laxatives, quality improvement project (qip)

## Abstract

Background: Constipation is one of the most common gastrointestinal problems affecting patients with frailty. Several factors in frail patients contribute to this issue, such as decreased mobility, low oral intake, comorbidities, polypharmacy, and susceptibility to drug side effects due to exhausted physiologic reserves. Constipation causes significant morbidity, including abdominal pain, overflow diarrhoea, urinary retention, delirium, and reduced quality of life; if not managed appropriately, it can lead to increased mortality. Therefore, timely recognition and a standardised, structured, stepwise approach are critical to preventing these outcomes. From anecdotal clinical experience in the frailty ward, while emphasis is placed on managing complex medical conditions, a comprehensive, standardised, and structured approach to managing constipation is sometimes overlooked, particularly among junior colleagues. This quality improvement project (QIP) aimed to improve compliance with national guidelines for managing frail patients with constipation by resident doctors.

Methodology: The QIP was conducted at Fairfield General Hospital in the frailty ward from March 2026 to August 2026, employing the Plan-Do-Study-Act (PDSA) methodology. The objective was to improve compliance with the national guidelines for the management of constipation in frail patients, focusing on key gaps identified in the baseline audit, namely the pertinent abdominal and per rectal (PR) exam, review of constipating medications, and first-line laxative prescribing.

Results: A baseline audit revealed an abdominal exam documentation rate of 33% (n=5); a review of constipating medications was performed 26% (n=4) of the time, and appropriate first-line laxatives were prescribed in 46% (n=7) of cases. Following this, targeted interventions such as ward-based teaching sessions as per national guidelines, drop-in sessions, and a six-point, user-friendly, standardised checklist were implemented over a period of two months. A retrospective cross-sectional study reviewing 15 case notes revealed marked improvements across all audited parameters: abdominal exam documentation improved to 86%, medication review to 80%, and first-line laxative prescribing to 80%.

Conclusion: Compliance with national standards in managing constipation in frail patients among newly rotated residents can be improved through teaching sessions focused on national best practice guidelines. Furthermore, the use of a user-friendly, standardised, and accessible checklist in our QIP was associated with improved compliance with best practices and enhanced quality of care.

## Introduction

Constipation is a heterogeneous, symptom-based syndrome with facets of presentation such as infrequent bowel motions or hard stools, difficulty passing stool, often with associated straining, and a sense of incomplete emptying [1]. Rome IV criteria require two or more of the following to diagnose constipation: straining, lumpy or hard stools, sensation of incomplete evacuation and sensation of anorectal obstruction or blockage in more than 25% of defecations, and fewer than three spontaneous bowel motions in a week [2]. However, in practice, particularly in the elderly, less frequent bowel motions from the patient’s normal often make the diagnosis [3]. Furthermore, in geriatric patients, constipation can present as confusion, overflow diarrhoea, or urinary retention [3], which necessitates thorough assessment and prompt management when these are identified. Constipation is quite common in frail patients although large scale nationwide epidemiologic studies are scarce: a research on constipation in elderly, which enrolled 439 geriatric inpatients, 183 care home residents, and 78 visitors, of a geriatric day hospital showed a constipation prevalence of 79% (n=347) in geriatric ward inpatients [4], while a study on the prevalence of faecal impaction in nursing homes, which sampled 487 residents, revealed a 70.7% (n=486) prevalence of constipation in care homes [5]. It is a significant contributor to and sequelae of the five aspects of frailty syndrome, touching on susceptibility to drug side effects, polypharmacy, decreased mobility, delirium, urinary retention/incontinence, and falls [6,7]. Hence, it is a significant cause of morbidity and mortality in frail patients, resulting in the above aspects of frailty syndrome and, in severe cases, syncopal episodes with resulting coronary and cerebral ischaemia from excessive straining [8]. To this end, prompt recognition through a standardised approach and early initiation of management per national guidance are vital for improving the quality of management of constipation and mitigating the above complications.

Frailty syndrome is a state of compromised physiologic reserve and functional decline due to multiple factors such as ageing, co-morbidities, and functional impairment, leading to vulnerability to stress, which otherwise is tolerable in a non-frail population and includes recognised facets of presentation such as falls, delirium, immobility, incontinence, and susceptibility to drug side effects [7].

A frailty ward is a medical ward designed to deal with complex medical and end-of-life issues for patients with frailty and significant comorbidity. Elderly patients are prone to developing constipation for the following reasons: compromised physiologic reserve increasing susceptibility to drug side effects, polypharmacy, decreased mobility, low oral intake, and compromised gastrointestinal function [9]. Focusing on dealing with complex medical issues in frail patients and the widespread prevalence of constipation in elderly patients sometimes leads to a substandard approach to the management of constipation in frail patients.

This quality improvement project (QIP) identified key gaps in the approach to frail patients with constipation and the step-wise management in the frailty ward, devised interventions such as a six-point, standardised checklist for residents, and demonstrated significant improvement in compliance with national guidelines [3,10] following implementation, which later were embedded in the routine processes of constipation management in the ward. 

Ward-based teaching sessions for resident doctors, informal drop-in sessions, and a six-point, user-friendly, standardised, national guidelines-aligned checklist were implemented over a period of two months, which led to the marked improvement.

## Materials and methods

Study design, period, and setting

This QIP employed a Plan-Do-Study-Act (PDSA) methodology with the aim of improving the assessment and management of constipation in resident doctors in line with national guidelines [10,11]. A special focus was placed on key gaps identified in the baseline audit, including pertinent abdominal and per rectal (PR) exams, review of constipating medications, and first-line laxative prescribing for frail patients in the geriatric ward. The primary objective of the project was to improve compliance with national guidelines regarding the approach to elderly patients with constipation. Our secondary objective was to evaluate the effectiveness of the interventions implemented, namely the checklist and teaching sessions. The project was conducted in the frailty ward at Fairfield General Hospital (Bury, UK) from March 2026 to August 2026, with the intervention implemented over a two-month period.

Two cycles of retrospective cross-sectional data collection were conducted, reviewing 15 randomly selected patients with constipation in the frailty ward, using a standardised proforma to assess compliance with each quality standard. Case notes were randomly selected using a random number generator from the list of patients with a documented diagnosis of constipation during the study period. The study population consisted of elderly medical patients (over the age of 65 years) diagnosed with constipation and admitted to the geriatric ward at Fairfield General Hospital.

Quality standards

The following quality standards were established in accordance with National Institute for Health and Care Excellence (NICE) guidelines: (i) Adults with constipation should have an assessment that includes a review of diet and lifestyle, a medication review, and a physical examination [10]. (ii) Adults with constipation should be offered a first-line laxative appropriate for their clinical situation [11]. The first-line laxative was defined as an osmotic laxative (e.g., macrogol or lactulose) as per NICE guidance for adults with constipation [11]. (iii) Constipating medications should be identified and reviewed at each prescribing encounter [11].

PDSA

Plan

Our group of three resident doctors convened as part of the 'Plan' to discuss this important aspect of frailty syndrome. We planned to conduct a baseline audit on parameters drawn from the national guidelines to identify gaps, and agreed to design interventions in response to the key gaps identified. This was followed by a baseline audit on 15 patients admitted to a frailty ward of 22 beds, utilising a standardised proforma developed from the project’s quality standards in accordance with NICE guidance on the assessment and management of constipation. This proforma included three vital areas: documentation (including the use of the Bristol Stool Chart [12] and bowel history documentation), clinical assessment (encompassing abdominal exams and PR examinations when indicated and constipating medication review), and management (covering provision of conservative advice and appropriate first-line laxative prescribing). Randomised sampling through a random number generator from the list of patients with a documented diagnosis of constipation in the geriatric ward was undertaken during the study period.

Do

This audit revealed key gaps as well as strengths in the management of constipation among elderly patients. There was a 100% (n=15) rate of daily reviews and use of the Bristol Stool Chart, with an 86% (n=13) rate of bowel history documentation, which was remarkable. However, there were significant gaps in compliance with national guidelines: only 33% (n=5) of patients’ notes showed documentation of abdominal exam, constipating medication review was done in only 26% (n=4) of patients, and appropriate first-line laxatives were prescribed in 46% (n=7) of the cases.

Following this study, the team met to brainstorm ideas for interventions to improve performance, focusing particularly on the identified key gap areas. A six-point, user-friendly, standardised checklist aligned with British Geriatrics Society (BGS) best practice principles and NICE guidance for assessing and managing constipation was designed, introduced to residents, and implemented for two months [10,13-15]. Additionally, two ward-based teaching sessions for residents working in the frailty ward were delivered, and several drop-in sessions regarding the QIP, interventions, and further plans were communicated.

Study

As part of the 'Study', after a couple of months of implementing the interventions, a cross-sectional retrospective data collection was conducted using the same standardised proforma as in the baseline audit. This involved reviewing 15 case notes, which revealed marked improvements across all studied parameters. Notably improved parameters included documentation of abdominal exams, which increased to 86% (n=13) from 33% (n=5), first-line laxative prescribing, which rose to 80% (n=12) from 46% (n=7), and review of constipating medications, which improved to 80% (n=12) from 26% (n=4).

Action

Subsequently, to complete the PDSA cycle, the aforementioned interventions, such as the checklist and drop-in sessions, were embedded in the routine process of addressing constipation in the frailty ward. The QIP was presented to the newly rotated resident doctors in the geriatric ward and at the hospital’s audit and governance meeting, and the project was registered with the hospital’s QIP department.

**Figure 1:**
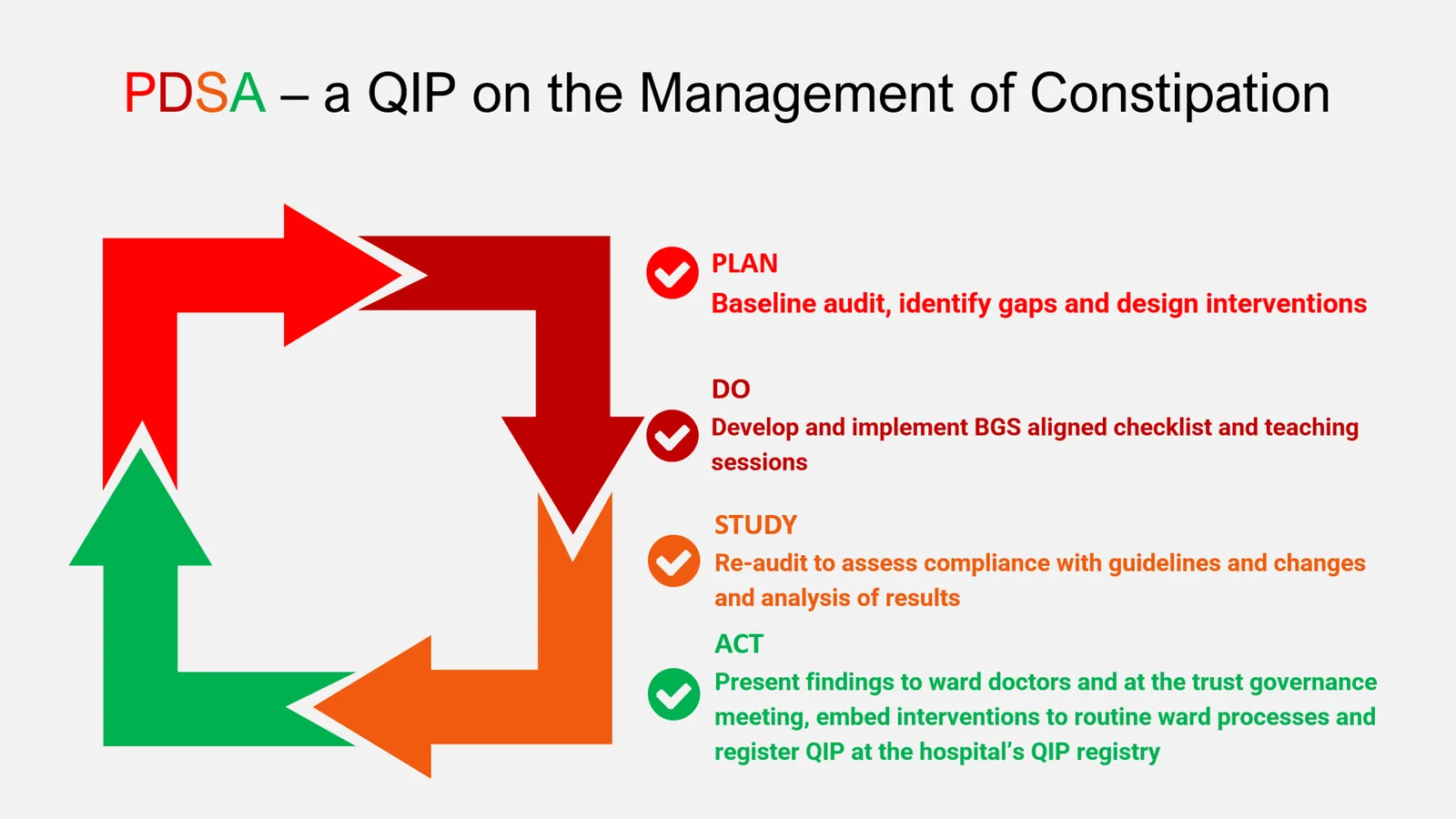
A summary of the PDSA methodology used in the project This figure details activities undertaken at each stage of the PDSA cycle of the project. BGS: British Geriatrics Society; PDSA: Plan-Do-Study-Action; QIP: quality improvement project Credits: Created by authors Yirdaw BG, Shaikh HA, and Adekiya V

Figure 1 depicts a summarised version of the PDSA methodology adopted in this project.

Interventions

Two forms of interventions were implemented, which brought about the desired changes.

Firstly, a six-point, user-friendly, standardised checklist aligned with national guidelines was designed, posted in the doctor’s office, and implemented during the two-month intervention period. This checklist was created based on the main points highlighted in national guidance, particularly BGS best practice principles and NICE guidelines, for the assessment and management of constipation [3,10,11,13-15], and it was approved by the geriatricians supervising care in the frailty ward.

The checklist provided quick prompts in the form of questions to assess compliance with national guidelines in the assessment and management of constipation. Accessibility was ensured by posting checklists on walls facing doctors as they used ward computers and through drop-in sessions that reminded staff about the checklist, its content, and where copies are posted on the wall.

Secondly, ward-based teaching sessions and informal drop-in sessions were delivered to resident doctors, including newly rotated foundation year 1 (FY1) doctors. All resident doctors working in the geriatric ward attended the two teaching sessions and several drop-in sessions that were planned in advance to ensure attendance.

The six-point, standardised checklist implemented and embedded in the routine processes of the geriatric ward is provided in Appendix A.

Strength and limitations

This project demonstrates strengths such as comprehensive coverage of multiple parameters, the use of a standardised checklist, and successful improvement in process measures, making it a valuable piece of work.

However, limitations such as the small overall sample size (n=15 per cycle), the single-centre design limiting generalisability, and the absence of checklist fidelity monitoring and sustainability data from further PDSA cycles are worth acknowledging.

## Results

The baseline audit conducted at the start of the project, which studied 15 case notes, revealed key gaps in the quality standards of the project, such as abdominal exam documentation at 33% (n=5), constipating medication review at 26% (n=4), and appropriate first-line medication prescribing at 46% (n=7). Furthermore, conservative advice provision was low, at a rate of 26% (n=4), and digital rectal examination (DRE) was performed in none of the three patients (0%) where it was indicated. DRE was considered indicated in patients with suspected faecal impaction, rectal bleeding, or where a rectal cause of constipation was suspected based on clinical assessment. Strengths identified from the first audit included a 100% (n=15) rate of Bristol Stool Chart use and daily patient reviews, with 86% (n=13) bowel history documentation.

**Figure 2:**
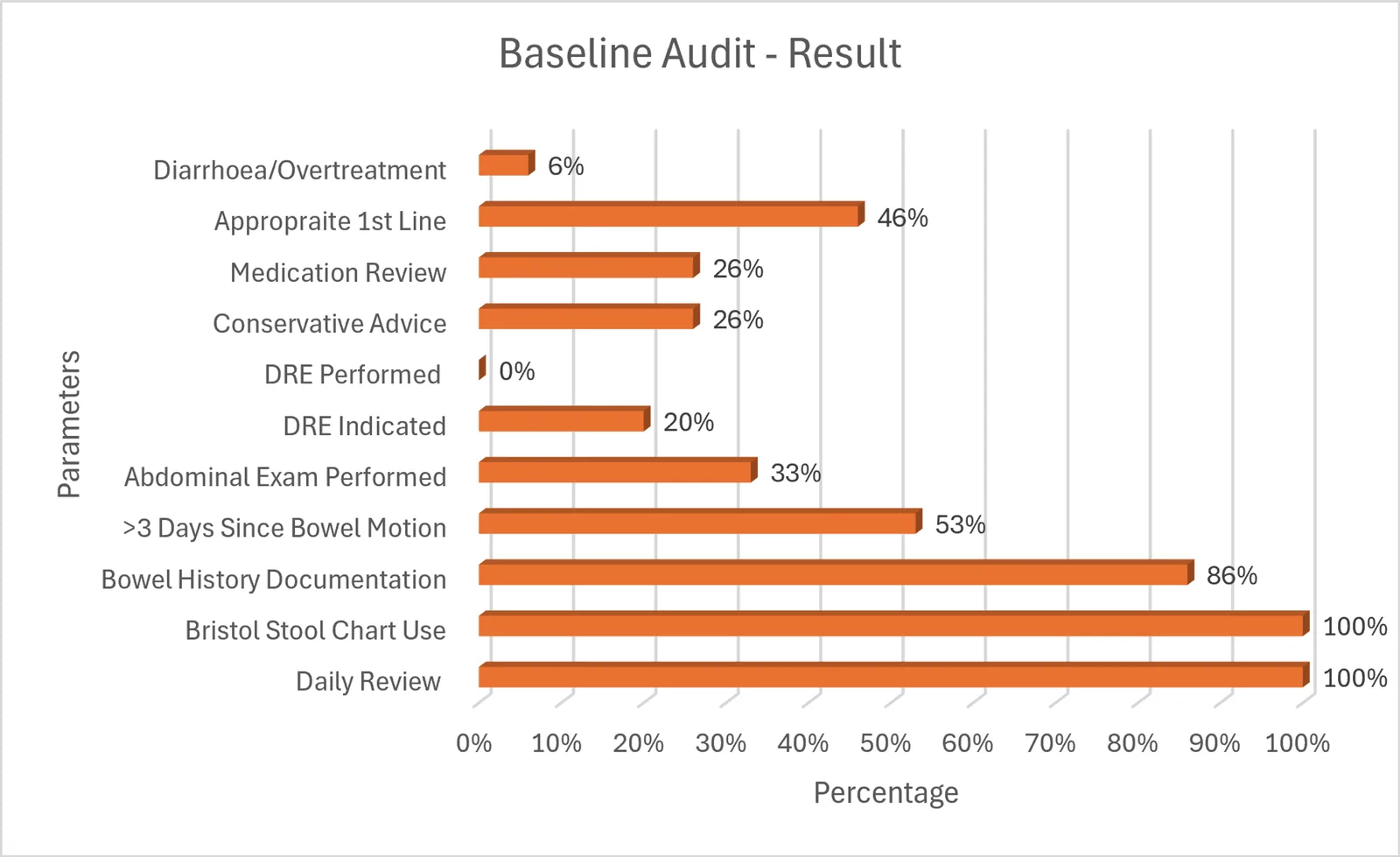
A bar graph showing the results of the baseline audit This graph details the results of the baseline audit conducted in the geriatric ward at Fairfield General Hospital. The audit assessed the parameters outlined above, focusing on documentation compliance, clinical assessment, and management of constipation among elderly patients. The parameters are plotted on the Y-axis, while the percentage of performance is plotted on the X-axis. Results are presented as percentages. There was a 100% (n=15) rate of daily reviews and Bristol Stool Chart use, with an 86% rate (n=13) of bowel history documentation. However, abdominal exam performance was recorded at 33% (n=5), with no per rectal exams performed, and a 26% rate (n=4) of constipating medication review. Furthermore, appropriate first-line laxatives were prescribed 46% (n=7) of the time, with conservative advice provision at 26% (n=4). DRE: digital rectal examination

Figure 2 demonstrates the findings of the baseline audit conducted in the geriatric ward.

Following a two-month period of intervention, marked improvements across all studied parameters were observed. Abdominal exam documentation increased to 86% (n=13), constipating medication review reached 80% (n=12), and a PR exam was performed in 60% (n=3) of the five cases with indications. Conservative advice was provided in 46% (n=7) of cases, and appropriate first-line laxative prescribing improved to 80% (n=12). 

**Figure 3:**
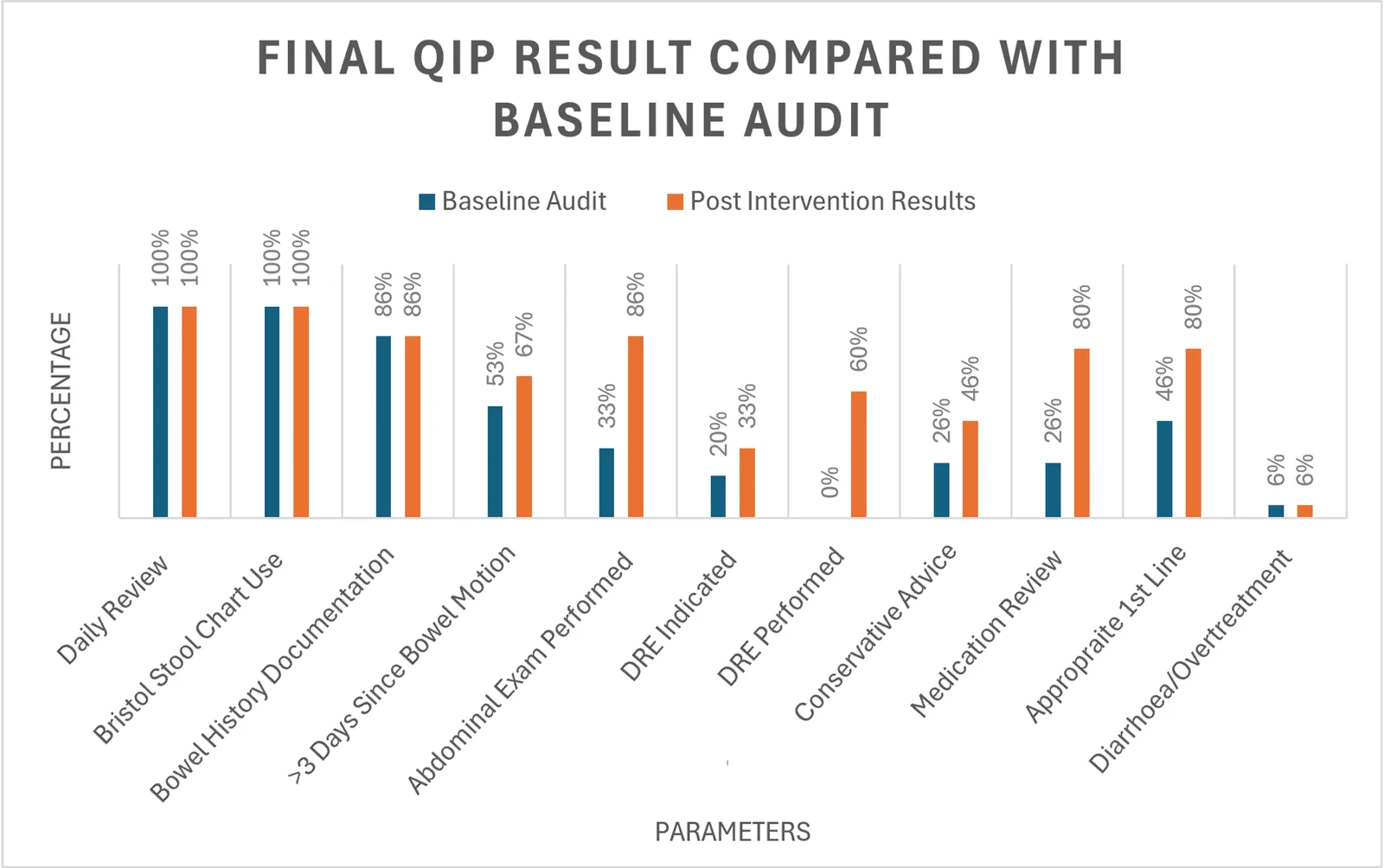
A comparative bar chart displaying the final result of the QIP in comparison to the baseline audit This graph shows the final results of the QIP in comparison with the baseline audit. The studied parameters are plotted on the Y-axis, while results are plotted on the X-axis in the form of percentages. Daily review remained at 100% (n=15), Bristol Stool Chart use at 100% (n=15), and bowel history documentation at 86% (n=13), all of which stayed high and consistent in the final stage of the PDSA cycle. There was marked improvement in abdominal exam documentation at 86% (n=13), constipating medication review at 80% (n=12), and appropriate first-line laxative prescribing at 80% (n=12). Furthermore, conservative advice provision had risen to 46% (n=7), and per rectal exam performance, when indicated, improved to 60% (n=3). QIP: quality improvement project; PDSA: Plan-Do-Study-Action; DRE: digital rectal examination

Figure 3 displays the results of the QIP following the implementation of interventions, highlighting the changes compared to the baseline audit.

**Figure 4:**
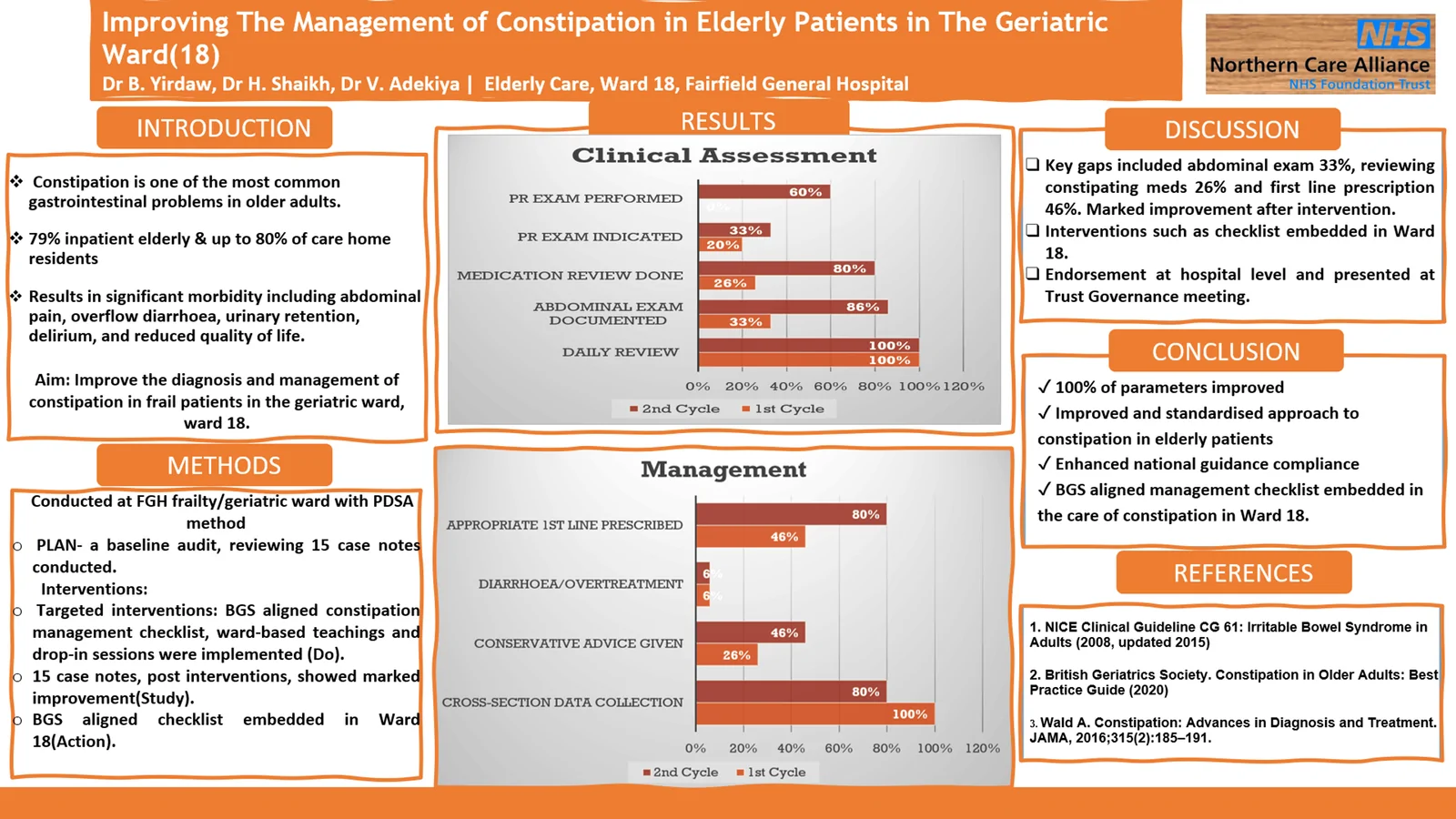
Poster of the QIP on improving the management of constipation in elderly patients in the geriatric ward at Fairfield General Hospital This poster summarises the QIP, detailing the introduction, methodology, and results of the project, along with a discussion of the results and conclusions drawn. QIP: quality improvement project; BGS: British Geriatrics Society; FGH: Fairfield General Hospital; PDSA: Plan-Do-Study-Action; PR: per rectal Credits: Created by authors Yirdaw BG, Shaikh HA, and Adekiya V

The project was presented at the hospital’s audit and governance meeting and registered with the QIP department with a poster. Figure 4 depicts the designed poster.

## Discussion

Constipation is a significant cause of morbidity in frail patients and can lead to serious complications such as delirium, falls, urinary retention, acute kidney injury, and, in severe cases, cerebral and coronary ischaemia from episodes of syncope due to straining if not managed properly [6,7].

The emphasis on complex medical issues in patients with frailty, the high prevalence of constipation among elderly inpatients, and the rotation of resident doctors contribute to substandard assessment and management of constipation in frail patients in geriatric wards. The BGS best practice principles and NICE guidelines outline the optimal management of constipation, including assessment, appropriate laxative prescribing, and review of constipating medications.

The baseline audit revealed gaps in documentation, clinical assessment, and management according to national guidelines. Key gaps identified included abdominal exam documentation at 33% (n=5), review of constipating medications at 26% (n=4), and appropriate first-line prescribing at 46% (n=7).

Following the implementation of targeted interventions, significant improvement in all studied parameters was demonstrated. For instance, abdominal exam documentation improved to 86% (n=13), appropriate first-line laxative prescribing rose to 80% (n=12), and review of constipating medications improved to 80% (n=12). DRE performance improved to 60% (n=3) from a null performance in the baseline audit. However, the PR exam findings were based on a very small number of indicated cases (five indicated cases, of which three were performed), limiting the generalizability of the improvement in this particular parameter. This project was limited to a single PDSA cycle; further cycles would be valuable to assess the sustainability of improvements and refine the checklist based on user feedback.

Several QIPs have been published regarding the improvement of assessment and management of constipation [16-20]. However, these projects focused on only a few parameters, while our QIP covered about eight important parameters, touching on history documentation, relevant physical examination, and comprehensive, standardised, and optimal management in line with the BGS best practice principles and NICE guidelines.

One project conducted in a gastroenterology ward with the aim of improving the recognition of constipation in predominantly elderly patients revealed a baseline Bristol Stool Chart use of 78.6% (n=19) and documentation in clinical notes of 17.5% (n=4), which aligns with our baseline audit showing abdominal exam documentation at 33% (n=5) but 100% Bristol Stool Chart usage (n=15) [16]. In that project, after several cycles of interventions reminding resident doctors, the stool chart monitoring - as documented in clinical notes - improved to 66.7% (n=16) from 17.5% (n=4) on the fourth PDSA cycle. Although this project demonstrated a similar improvement in documentation of bowel history, our project addressed a wider range of parameters, including relevant examinations, review of constipating medications, appropriate first-line laxative prescribing, and provision of conservative advice.

Another QIP on constipation management in a geriatric ward concluded that constipation management for elderly inpatients in that ward was inconsistent and suboptimal [17]. This retrospective data study of 50 patients revealed that documentation of individual bowel habits was 6% (n=3), daily bowel charting was at 53% (n=26), and faecal impaction checked by DRE was at 8.3%. Additionally, there was a 50% (n=25) rate of appropriate first-line laxative prescribing. The project proposed actions such as advocating for trust-based guidelines, staff training, flagging patients in board rounds, and promoting timely PR exams; with this, the project was closed. The results of this project mirror our baseline audit, which showed 0% for PR exams and 46% (n=7) for appropriate first-line laxative prescribing. However, daily bowel charting was quite low compared to our project, which showed 100% (n=15) use of the Bristol Stool Chart. Moreover, this project was halted with recommendations to improve identified problems, requiring interventions, and further study to complete the cycle. However, the project is a remarkable piece of work, which could serve as a foundation to complete the cycle.

One other project conducted in a geriatrics rehabilitation unit to improve the management of constipation in elderly inpatients, using a care bundle with a special focus on laxatives such as first-line laxative use, reducing pro re nata (PRN) laxatives, and using alternative high-fibre and fluid regimens for those who didn’t tolerate laxatives, revealed improved compliance with hospital-based guidance on laxative prescribing [18]. This project fine-tuned its focus on laxatives for managing constipation, while our project covered several parameters. However, this project completed two PDSA cycles and employed devised prescription guidance, bi-weekly laxative rounds, and the use of a poster, making it valuable work. Similarly, our project used ward-based teaching sessions, drop-in briefings, and a standardised checklist.

An additional QIP conducted at Manchester University NHS Foundation Trust on laxative prescription monitoring for constipated hospitalised adults identified gaps in appropriate laxative prescriptions and monitoring. The project designed and introduced a constipation management algorithm and provided education for staff, after which a marked improvement was noted [19]. This project focused on rationalising laxative prescriptions, reviewing laxatives once a week, and monitoring outcomes. The baseline study showed a 17% (n=8) rate of medication reviews once a week, a 28% (n=13) rate of mentions of laxative reviews at any point in the clinical notes, and a 52% (n=24) rate of documentation of rationale for prescriptions. Following the interventions, after six months, the review of weekly medication reviews rose to 83% (n=60), the documentation of laxative reviews increased to 81% (n=58), and the documented diagnosis of constipation reached 97% (n=70). Our project is similar in that a checklist was introduced as an intervention, laxative prescriptions were audited, and sustained improvement was demonstrated. However, this project contrasts in that it focused solely on monitoring laxatives and studied a larger sample. Moreover, the QIP informed the trust's policy, where the introduced algorithm was incorporated.

The final project under consideration was a study conducted in the geriatric ward of Cambridge University Teaching Hospitals, which focused on improving the recognition of constipation through better use of stool charts [20]. A baseline audit revealed a stool chart usage rate of 92% (n=25), good quality documentation of bowel history at 76% (n=19), and a PR exam performance of 0%. Interventions included encouraging staff to review charts, using smart links to access and import charts, and highlighting incomplete documentation to ward staff. The third audit, after these interventions, showed stool chart usage of 93% (n=25), good quality bowel motion tracking at 80% (n=20), and a persistent 0% rate for PR exams. Our project is broader in terms of covering a wider array of parameters related to documentation, assessment, and management. The PR exams in this project remained at nil, while our project saw an increase to 60% (n=3) pre-enema following interventions. This project is useful in identifying both gaps and strengths within the geriatric ward at the hospital.

This QIP, under discussion, is unique in that it covers a comprehensive set of eight parameters spanning documentation, clinical examination, and management, whereas most previous QIPs focused on a narrower set of outcomes, making this project both unique and valuable.

A research article titled "Understanding Constipation as a Geriatric Syndrome" argues for treating constipation as a geriatric syndrome due to its high prevalence resulting from age-related changes and the predisposing and precipitating factors in elderly patients [21]. The article recommends inquiring about bowel movements and constipation symptoms in geriatric patients using a stepwise standardised approach to managing constipation in this cohort. This research aligns with our QIP in that it includes a literature review highlighting the high prevalence of constipation in geriatric patients and implements interventions to improve the approach to constipation in elderly patients according to national guidelines. The project demonstrated significant improvements in managing constipation through teaching sessions and a management checklist aligned with national guidelines.

Another study on chronic constipation in the elderly provided a thorough update on evaluating and managing constipation, detailing a stepwise approach in assessment and management for elderly patients [2]. This study emphasised the importance of thorough history-taking that focuses on alarm signs, past medical history, and medication use. Additionally, examinations including perianal exam, digital rectal exam, and relevant blood tests such as full blood count (FBC), renal, liver, and thyroid function tests, and inflammatory markers were emphasised. While specialist physiologic tests such as evacuation proctography, radiopaque markers, and anorectal manometry with wireless motility capsules are rarely needed in elderly patients, their use is warranted in refractory cases or when there's suspicion of defecatory disorders [2]. Management, as per the study, begins with conservative measures in mild cases, which include increased oral fluid intake, dietary fibre (starting at 5 grams and titrated up to 25-30 grams), and increased moderate activity along with appropriate toilet education. The study promotes osmotic laxatives as the first line of treatment, followed by stimulants if no adequate response is achieved [2]. Lastly, biofeedback sessions were shown to be effective in elderly patients with pelvic floor dyssynergia [2]. This study aligns with national guidelines and goes hand-in-hand with the QIP under discussion in that a stepwise approach to managing constipation is emphasised.

A systematic review on managing constipation in the elderly evaluated 23 randomised controlled trials that investigated the effectiveness and safety of laxatives in this patient cohort, making comparisons between laxative use and placebo, among the laxatives within the same group and across different groups [22]. Four placebo-controlled trials on bulk-forming laxatives did not show a marked increase in bowel movements, although transit time and stool consistency were demonstrated to improve [22]. Furthermore, these trials reviewed a limited number of cases, with two covering fewer than 10 patients.

Seven randomised controlled trials on osmotic laxatives were reviewed, four of which compared osmotics with placebo and three of which contrasted different osmotic laxatives [22]. In the placebo-controlled trials, a significant increase in bowel movements, a decreased need for laxatives, and improved stool consistency were demonstrated [22]. Two studies comparing osmotic laxatives (lactulose vs. sorbitol and polyethylene glycol (PEG) 4000 without and with electrolytes) revealed negligible differences in terms of stool consistency or bowel movements [22]. These findings concur with national guidelines recommending osmotic laxatives as the first-line treatment for elderly patients and support the interventions of this project.

## Conclusions

Emphasis on complex medical issues in frail patients, the high prevalence of constipation among elderly inpatients, and the rotation of staffing in the geriatric ward could lead to suboptimal management of constipation in this cohort. A baseline audit revealed inconsistent documentation and substandard assessment and management of constipation in the geriatric ward. However, following a two-month period of intervention that included teaching sessions and the introduction of a standardised checklist, a marked improvement in the assessment and management of constipation was noted. Further PDSA cycles are needed to assess the sustainability of these improvements beyond the initial two-month intervention period. Embedding routine teaching sessions for newly rotated resident doctors and utilising a standardised checklist aligned with national guidelines in the process of managing constipation is associated with the improvement of overall assessment and management of constipation in frail patients admitted to geriatric wards.

## Figures and Tables

**Figure 5:**
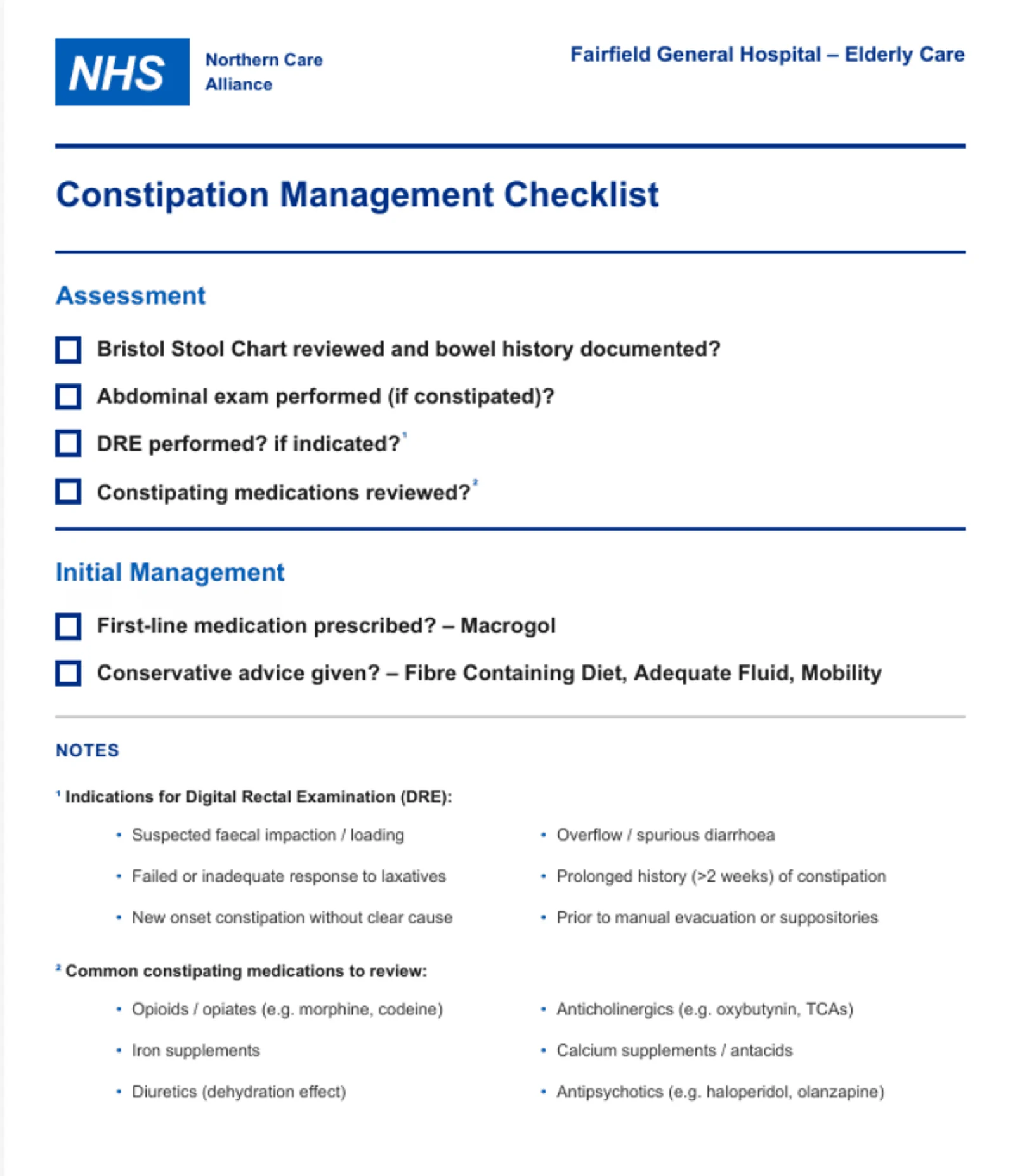
The six-point, national guideline-aligned constipation assessment and management checklist introduced in the QIP This checklist was designed in accordance with national guidelines and implemented to improve the assessment and management of constipation among elderly patients in the geriatric ward at Fairfield General Hospital. QIP: quality improvement project; TCAs: tricyclic antidepressants Credits: Created by authors Yirdaw BG, Shaikh HA, and Adekiya V

## Appendices

Appendix A
